# Abusive Supervision, Leader-Member Exchange, and Creativity: A Multilevel Examination

**DOI:** 10.3389/fpsyg.2021.647169

**Published:** 2021-05-05

**Authors:** Changqing He, Rongrong Teng, Liying Zhou, Valerie Lynette Wang, Jing Yuan

**Affiliations:** ^1^College of Economics and Management, Nanjing University of Aeronautics and Astronautics, Nanjing, China; ^2^School of Business, Guizhou University of Finance and Economics, Guiyang, China; ^3^College of Business and Public Management, West Chester University, West Chester, PA, United States; ^4^College Students Quality Education Research Center, Anhui Xinhua University, Hefei, China

**Keywords:** abusive supervision, team creativity, individual creativity, team-level leader-member exchange, LMX differentiation

## Abstract

Despite the growing attention on the topic of abusive supervision, how abusive supervision affects individual and team creativity have not yet been thoroughly investigated. Drawn from the perspective of leader-member exchange (LMX), the current study develops a multilevel model to describe the relationships between abusive supervision and creativity at both team and individual levels, with a focus on the roles played by team-level leader-member exchange (TLMX) and LMX differentiation (DLMX). Based on data collected from 319 team members and their team leaders in 71 teams, the results show that abusive supervision has a negative relationship with TLMX, a practice that is conducive to both team and individual creativity. At the team level, the negative relationship between abusive supervision and TLMX is lessened by a higher level of DLMX. In addition, the positive relationship between TLMX and team creativity is weakened by a higher level of DLMX. Theoretical and practical implications of the findings are discussed.

## Introduction

In today’s competitive and rapidly changing business environment, scholars are increasingly interested in understanding how to enhance creativity in organizations (Shalley et al., 2004, 2009). One vein of research attempts to describe the relationship between abusive supervision and creativity (Liu et al., 2012; Lee et al., 2013; Shen et al., in press), and focuses on the impact of abusive supervision on creativity at the individual employee level (Liu et al., 2012, 2016; Zhang et al., 2014; Gu et al., 2016; Shen et al., 2020).

However, the relationship between abusive supervision and creativity at the team level has received less attention. Team creativity is defined as the generation of novel and useful ideas by employees working together in a team (Shin and Zhou, 2007). Previous studies have highlighted the positive aspect of leadership, such as transformational leadership, in promoting team creativity (Bai et al., 2016). As a pervasive leadership style, abusive supervision may also play a critical role in shaping team members’ creative performance (Liu et al., 2012). Yet, the effects of abusive supervision on team creativity remain largely unexplored.

Furthermore, an even more important question is how team-level abusive supervision affects team creativity and individual creativity simultaneously. Previous findings on the effects of abusive supervision on creativity at the individual level are limited and inconsistent, with some studies reporting negative relationships (Liu et al., 2012; Zhang et al., 2014), and others suggesting a curvilinear relationship (Lee et al., 2013). Moreover, although a number of mediators and moderators have been identified to explain how abusive supervision influences creativity (Gu et al., 2016; Liu et al., 2016), the mechanism of how abusive supervision affects creativity at both team and individual levels needs state-of-the-art deliberation. This study aims to fill this gap by investigating the mediating and moderating mechanisms through which abusive supervision affects creativity at the two different levels.

The leader-member exchange (LMX) perspective has been used to examine motivation and social exchange processes that can facilitate creativity (Pan et al., 2012; Xu et al., 2012). Although LMX was initially conceptualized in a dyadic pattern (e.g., a supervisor and a subordinate), previous research has found that LMX occurs at multiple levels, including the team level and the individual level (Boies and Howell, 2006; Li and Liao, 2014). In particular, team-level LMX (TLMX) was operationalized as the mean score of team members’ ratings of their relationship with the team leader, reflecting the extent to which exchange is carried out between the entire team and the team leader (Boies and Howell, 2006). From this perspective, we expect that abusive supervision affects individual and team creativity through the mediation of TLMX.

Additionally, one of the premises of LMX theory is that the exchange patterns differ between a leader and the subordinates (Liden et al., 2006). LMX differentiation (DLMX) represents the variation in the quality of the exchange relationships between the team leader and the team members (Erdogan and Liden, 2002). It facilitates or hinders individual motivation and team motivation for social exchange associated with abusive supervision (Li and Liao, 2014). Thus, it has critical implications for both individual and team performance outcomes (Kauppila, 2016; Sui et al., 2016; Martin et al., 2018). We expect that DLMX plays a moderating role in the relationships among abusive supervision, TLMX, and creativity.

**FIGURE 1:**
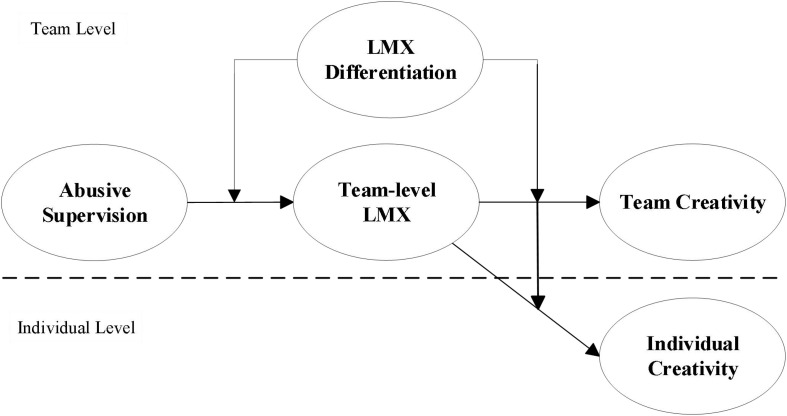
Hypothesized research model.

To this end, we adopt Input-Mediator-Outcome (IMO) Model that describes a team as a system, which relies on various team inputs through intermediate interactive processes to produce team outcomes (Mathieu et al., 2008; Hu et al., 2020). Abusive supervision is considered as a key input that shapes team process in terms of TLMX between the team leader and the team members (Andressen et al., 2012). We argue that, through the mediation of TLMX, the input influences performance outcomes, including team creativity and individual creativity. Further, we propose that DLMX moderates the relationship between abusive supervision, TLMX, and creativity. The research framework is depicted in Figure 1.

## Theoretical Framework and Hypotheses

### Abusive Supervision, Team-Level Leader-Member Exchange, and Creativity

#### Abusive Supervision and Team-Level Leader-Member Exchange

Abusive supervision is understood as team members’ shared understanding about their team leader’s negative actions toward the team (Murase et al., 2014). That is to say, all the team members collectively believe that their leader will exhibit particular negative gestures that can occur at any time to any team member (e.g., depreciating team members’ thoughts or feelings) (Tepper, 2007). On the other hand, team-level leader-member exchange (TLMX) represents the full spectrum of social exchange relationships between the team leader and the team members that are developed over time (Boies and Howell, 2006). Thus, the team members experiencing the team leader’s abusive behavior will report low TLMX. However, the two constructs (i.e., abusive supervision and TLMX) are conceptually distinctive from each other (Xu et al., 2012). Consistent with prior research (Lian et al., 2012; Xu et al., 2015; Haggard and Park, 2018), we consider abusive supervision an antecedent that negatively impacts TLMX.

According to LMX theory, the supervisor and the subordinates develop their exchange relationships through a role-defining process, in which they experiment with each other on the basis of certain role expectations (Graen, 1976; Xu et al., 2012). When they find that the other party meets the expectations, they tend to pursue a higher level of LMX. Under abusive supervision, the development of high-quality LMX between the team leader and the team members is likely to be impeded.

Furthermore, team members who experience team leader’s abusive behavior are more likely to exhibit deviance (Tepper et al., 2009; Javed et al., 2019) and directed destructive voice (Mackey et al., 2020) toward the team leader, and are less likely to engage in positive behavior, such as OCB (Xu et al., 2012) and personal initiatives (Pan and Lin, 2018). In return, these behaviors become negative feedback to the team leader, leading to circumstances in which the team members appear underneath the team leader’s expectations. Thus, the team leader and the team members are less likely to develop a high level of TLMX as a result of abusive supervision (Xu et al., 2012; Haggard and Park, 2018).

#### Team-Level Leader-Member Exchange and Creativity

High levels of TLMX can facilitate employee creativity in several ways. First, under high TLMX, both the whole team and individual team members are more likely to receive valued resources and strong support from the team leader, which are crucial for the inception of creativity (Lin et al., 2018). For example, in high-quality exchange relationships, the team leader shares more constructive and more comprehensive ideas with the team members. Enhanced knowledge sharing will help both the team and individual employees in the team achieve higher levels of creativity (Oldham and Cummings, 1996).

Second, team members who experience high-quality exchange relationships with their team leader are more motivated and more likely to enjoy autonomy in dealing with challenging tasks (Lin et al., 2018; Kirrane et al., 2019). Risk taking in new procedures and experimenting with novel ideas lead to superior creativity for the whole team and the team members.

Taken together, following the IMO Model, we propose that TLMX will mediate the relationships between abusive supervision and creativity at both team and individual levels. According to Tierney et al. (1999), TLMX is a key process linking leadership with employee creativity. Abusive supervision will impair TLMX, which corresponds to the social exchange between the team leader and the team members. In turn, lower TLMX leads to reduced creative activities for the team as well as for team members due to the lack of support and valued resources to conduct creative work. To summarize, we propose two mediated relationships:

**Hypothesis 1a**: Abusive supervision has an indirect negative relationship with team creativity via team-level leader-member exchange.

**Hypothesis 1b**: Abusive supervision has an indirect negative relationship with individual creativity via team-level leader-member exchange.

### Moderating Role of LMX Differentiation

To better explain the mechanism of TLMX, it is important to consider the nuanced difference in dyadic relationships between the team leader and the team members. LMX differentiation (DLMX) can help explain why the effects of abusive supervision on TLMX are stronger for some teams. Previous research has revealed that a team leader’s influence on team members’ performance strongly depends on how the team leader interacts with each and every team member (Wu et al., 2010; Mackey et al., 2020). In view of this logic, we consider DLMX a key moderator of the relationship between abusive supervision and TLMX.

More specifically, we argue that higher DLMX will lessen the negative relationship between abusive supervision and TLMX. First, DLMX will buffer the process of role expectations linking abusive supervision and TLMX. As discussed above, abusive supervision will hinder TLMX because the abusive behavior displayed by the team leader does not meet the role expectations of the team members. Under high DLMX, the team leader treats team members differently and only rewards those members who are the most developed, competent, and skilled (Carnevale et al., 2019). The team members can reasonably anticipate that the team leader is not going to back up other team members. Thus, the team members tend to view differentiated leadership more favorably even though the team leader displays occasional abusive behavior toward them (Carnevale et al., 2019).

**FIGURE 2:**
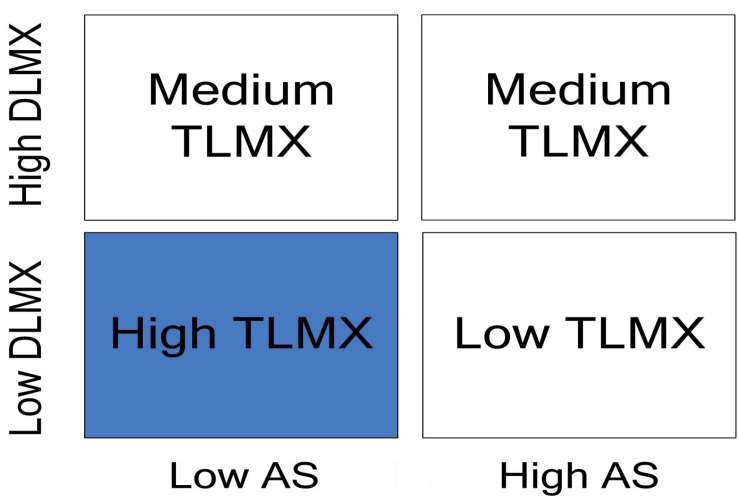
Hypothesized interaction effects of abusive supervision and DLMX on TLMX.

Second, the buffering mechanism taken by DLMX can be explained by the substitution effect or the crowding-out effect (Cai et al., 2019). When DLMX is higher, team members tend to perceive lower equality, which is also an outcome of higher abusive supervision. Thus, we expect to see a slower decrease in TLMX as abusive supervision increases within higher levels of DLMX (see Figure 2). Therefore, we offer the following hypothesis:

**Hypothesis 2**: LMX differentiation moderates the relationship between abusive supervision and team-level LMX, such that this negative relationship is weakened when LMX differentiation is higher.

From a social comparison perspective, we propose that DLMX operates as a social cue driving team members to develop justice perceptions, which ultimately weaken the positive relationship between TLMX and creativity at both team and individual levels. High DLMX means that a team leader only provides abundant resources to some employees instead of all the team members. In a team with higher levels of DLMX, the perceptions of justice and equality are likely to be questioned by the team members, who tend to exhibit decreased motivation levels in creative activities (Harris et al., 2014). In contrast, in a team with lower levels of DLMX, each team member experience similar leader-member exchange relationships with each other (Carnevale et al., 2019). Support from the team leader and the distribution of resources tend to be consistent across team members (Carnevale et al., 2019). The justice and equality perceived by team members are conducive to engaging in creative activities. Accordingly, both the whole team and the individual team members are more likely to benefit from TLMX and achieve planned outcomes from creative activities under lower levels of DLMX.

Another reason for the attenuating effects of DLMX comes from social identity theory. Koh et al. (2019) argued that team members are more likely to engage in creative activities when the contextual factors help them build a favorable identity. When DLMX is lower, team members are more likely to associate themselves with the overall team identity (Harris et al., 2014). Therefore, low levels of DLMX will facilitate the positive relationship between TLMX and creativity. On the contrary, when DLMX is high, team members cannot easily label themselves as an integral part of the team. As a result, the positive relationships between TLMX and creativity at both team and individual levels are hindered. Therefore, we offer the following two hypotheses:

**Hypothesis 3a**: LMX differentiation moderates the relationship between team-level LMX and team creativity, such that this positive relationship is lessened when LMX differentiation is higher.

**Hypothesis 3b**: LMX differentiation moderates the relationship between team-level LMX and individual creativity, such that this positive relationship is lessened when LMX differentiation is higher.

## Materials and Methods

### Sample and Procedures

Data collection was conducted in Central China using the snowball approach. Data for this study were obtained from working teams in a number of industries, including manufacturing, technology, R&D, service and marketing. A work team was defined as two or more interdependent individuals who work jointly to accomplish common goals (Le Blanc et al., 2021). The snowball data collection approach is particularly useful in China, where *guanxi* (or personal relationships) significantly facilitates access to critical information (Easterby-Smith and Malina, 1999). Through the snowball data collection approach, we identified 86 team leaders in different industry sectors and requested their participation in this study. After obtaining their permission, we asked the team leaders to invite their employees to participate in this study. We used a coding scheme to give each participant a unique code to guarantee the confidentiality of all employee responses.

To test our conceptual model (see Figure 1), we sought to collect data from multiple team members within each team to reduce potential single-rater bias. A paper-and-pencil survey package was distributed to both the team leader and the team members in each participating team at the same time. Each survey package contained two separate questionnaires, one for the team leader and the other one for the team members. A cover letter attached to each questionnaire explained the objective of the survey and assured respondents of the confidentiality of their responses. The team leader questionnaire contained questions on team creativity, team size and team tenure. The team members questionnaire asked team members to assess abusive supervision, LMX, and individual creativity. Completed questionnaires were returned to the researchers in the sealed package.

Of the 86 survey packages distributed, 81 were returned, representing a response rate of 94.19%. For a team to be included in the final sample, the team leader and at least three team members had to complete the questionnaire (Somech et al., 2009). Data with missing values were discarded. Finally, data from 71 teams were usable for the statistical analysis. The data set comprised 71 team leaders and 319 team members.

The final sample consisted of manufacturing teams (28.2%), R&D teams (33.8%), marketing teams (5.6%), and others (32.4%). The diverse background of teams in this sample helps establish the generalizability of the findings (Wang and Rode, 2010). Based on team size, teams in the sample were categorized into 1–5 members (4.2%), 6–10 members (42.3%), 11–15 members (12.7%), and 16 members and above (40.8%). ANOVA was used to test whether team responses significantly differ by team size. The results showed that none of the variables exhibited significant differences. Team tenure categories included 1 year and below (2.8%), 1–2 years (16.9%), 2–5 years (21.1%), 5–10 years (39.4%), and 10 years and above (19.7%). The team leaders were primarily men (84.8%). The age groups of the team leaders included 21–30 years (20.7%), 31–40 years (45.3%), 41–50 years (30.2%), and 50 years and above (3.8%). The team members were also primarily men (62.4%). The age groups of the team members included 21–30 (59.5%), 31–40 years (34.7%), 41–50 years (5.1%), and 50 years and above (0.7%).

### Measures

All the measures used in this study were adapted from existing literature originally written in English language. A number of necessary steps were taken to ensure that the measures were appropriately translated and phrased for Chinese employees. We followed the standard translation-back-translation procedure recommended by Brislin (1970, 1990). A researcher who is fluent in Chinese translated existing English language measures into Chinese. Two Chinese professors who are proficient in English improved the translation through iterative processes where any concerns or discrepancies between the English and Chinese versions were detected and addressed. To validate the survey translation, three Chinese employees not affiliated with this study read through the Chinese version to test its readability and ease of comprehension. Remaining concerns were noted and addressed. As a final check, a Chinese native translated the survey back into English, and the Chinese and English versions were compared for any major discrepancies. The final version of the questionnaire containing the translated measures was adopted when no major discrepancies were found. All the measures were built on a 5-point Likert-type scale.

#### Abusive Supervision

Abusive supervision was measured by a 15-item scale developed by Tepper (2000). Using this measure, team members assess the frequency of abusive behavior of their team leaders. An example item states, “My leader tells me my thoughts or feelings are stupid” (1 = never, 5 = very frequently). Cronbach’s alpha was 0.931 for abusive supervision.

#### TLMX and DLMX

TLMX is calculated as the mean of all the participating team member’s ratings on the quality of the relationship with the team leader (Boies and Howell, 2006). Individual-level LMX was assessed using the 7-item LMX scale developed by Graen and Uhl-Bien (1995). An example item states, “I would characterize my working relationship with my leader as extremely effective” (1 = strongly disagree, 5 = strongly agree). Cronbach’s alpha was 0.888 for LMX. Consistent with previous DLMX measures (Harris et al., 2014), we used the variance in the individual-level LMX scores in each team to measure LMX differentiation. Higher within-team variance reflects higher LMX differentiation.

#### Individual Creativity

We used Farmer et al. (2003)’s 4-item creativity scale that has been used for Chinese employees. One sample item reads, “I seek new ideas and ways to solve problems” (1 = strongly disagree, 5 = strongly agree). Cronbach’s alpha was 0.830 for individual creativity.

#### Team Creativity

Team creativity was measured by four items from Shin and Zhou (2007). These items measured the extent to which a team produces novel and useful ideas. Consistent with previous studies (Ali et al., 2020), we ask team leader to report team creativity. Using a 5-point scale (1 = poorly; 5 = very much), the team leader rated the team in questions such as “How well does your team produce new ideas?” Cronbach’s alpha value was 0.889.

#### Control Variables

We controlled for alternative explanations by including individual and team level control variables. The literature suggested controlling for gender, age, and education level at the individual level (Amabile, 1988), and team’s tenure and size at the team level (Harrison et al., 2002).

### Statistical Analysis

Given the multilevel nature of the data, we conducted hierarchical linear modeling (HLM) using HLM 6.02 to test the hypotheses. For cross-level relationships, we used two-level models with team members at level 1 and teams/leaders at level 2. To test the mediating role of TLMX, we applied the product of coefficients test recommended by previous research (MacKinnon et al., 2004).

In addition, we tested within-team agreement for abusive supervision and TLMX by computing with-group interrater agreement (R_wg_). The mean values of R_wg_ for abusive supervision and TLMX were 0.921 and 0.905, respectively. After high levels of mean R_wg_ were identified for abusive supervision (0.921, range = 0.56–1) and TLMX (0.905, range = 0.51–1), we attempted to find how many teams had low R_wg_. We found that 92% of the teams on abusive supervision and 93% of the teams on TLMX had an R_wg_ value higher than the 0.70 criterion (Gong et al., 2013). In addition, we examined between-group variability by calculating intra-class correlation ICC(1), and reliability of the mean ICC(2). The results supported the aggregation of abusive supervision to a team-level variable [ICC(1) = 0.230, ICC(2) = 0.622]. Meanwhile, ICC(1) was 0.250 and ICC(2) was 0.647 for TLMX. These values exceeded the levels for aggregation recommended by prior research (Gong et al., 2013). Therefore, we aggregated team members’ ratings of abusive supervision and LMX to the team level.

## Results

### Descriptive Statistics

Table 1 shows the means, standard deviations, inter-correlations, and reliability coefficients of all the variables.

**TABLE 1:** Means, standard deviations, and correlations.

Variables	Mean	SD	1	2	3	4	5	6	7
**Individual-level variables**									
1. Gender	–	–							
2. Age	–	–	0.102						
3. Education level	–	–	0.008	−0.189**					
4. LMX	3.729	0.546	0.073	–0.034	–0.069	(0.888)			
5. Individual creativity	3.832	0.573	0.175**	–0.024	–0.001	0.423***	(0.830)		
**Team-level variables**									
1. Team size	–	–							
2. Team tenure	–	–	0.026						
3. Abusive supervision	1.277	0.395	0.001	0.018					
4. TLMX	3.728	0.344	0.038	0.010	−0.475***				
5. DLMX	0.368	0.235	–0.146	–0.029	0.280*	−0.375**			
6. Team creativity	3.603	0.408	0.163	–0.080	−0.159*	0.511***	−0.276*	(0.889)	

N (subordinate) = 319, N (leader/team) = 71. Reliabilities are in parentheses on the diagonal. *p < 0.05; **p < 0.01; ***p < 0.001.

### Confirmatory Factor Analysis (CFA)

We conducted confirmatory factor analysis (CFA) to test the distinctiveness of the three self-reported variables, abusive supervision, LMX, and individual creativity. To achieve an optimal ratio of sample size to number of estimated parameters and avoid testing a too complex model, we combined items to create three indicators for each construct (Landis et al., 2000; Zhang et al., 2012). We developed a baseline three-factor model and four alternative models and then tested Chi-square differences to see which model was better (Anderson and Gerbing, 1988). As shown in Table 2, the hypothesized three-factor model exhibited a better fit to the data [χ^2^ = 39.51, *df* = 24, *p* < 0.001, RMSEA = 0.045, SRMR = 0.028, CFI = 0.99, NFI = 0.97, NNFI = 0.99] and had a significantly better fit than all of the four alternative models. Moreover, in our hypothesized three-factor model, all loadings were significant on their respective factors. The results suggested that the variables had satisfactory discriminant validity.

**TABLE 2:** Model fit indices for confirmatory factor analysis.

Model	χ ^2^	df	Δχ ^2^ (Δ df)	RMSEA	SRMR	CFI	NFI	NNFI
1. Hypothesized three-factor model	39.51	24	–	0.045	0.028	0.99	0.97	0.99
2. Two-factor model (LMX and CR are combined)	293.92	26	254.41 (2)	0.178	0.110	0.89	0.88	0.84
3. Two-factor model (AS and CR are combined)	455.39	26	415.88 (2)	0.226	0.180	0.83	0.82	0.77
4. Two-factor model (AS and LMX are combined)	652.87	26	613.36 (2)	0.273	0.190	0.74	0.73	0.64
5. Single-factor model	989.01	27	949.50 (3)	0.332	0.200	0.55	0.54	0.40

**N = 319. In calculating the correlations, we disaggregated the value of team member ratings of abusive supervision and LMX (TLMX/DLMX) to individual level. AS, abusive supervision; LMX, leader-member exchange; CR, individual creativity; RMSEA, root mean square error of approximation; SRMR, standardized root square residual; CFI, comparative fit index; NFI, Normed fit index; NNFI, Non-Normed fit index. All Δχ2 are significant at p < 0.001.**

### Common Method Variance

Although we employed a multi-source survey to measure our variables (i.e., leaders and subordinates), team members’ ratings of abusive supervision, LMX, and creativity may suffer from common method bias. We applied Harman’s one-factor test (Podsakoff and Organ, 1986; Podsakoff et al., 2012) to examine possible common method variance. Three factors that account for 62.53% of variance were extracted and the first factor accounted for 26.08%. Thus, common method variance was not concern for our results.

### Hypothesis Testing

Hypothesis 1a proposed that abusive supervision has an indirect negative relationship with team creativity through TLMX. Results showed that abusive supervision was significantly related to TLMX (β = −0.476, *p* < 0.001; see Model 1 in Table 3). TLMX was significantly related to team creativity (β = 0.506, *p* < 0.001; see Model 5 in Table 3). The bootstrapping test based on MacKinnon et al. (2004) procedure indicated that the indirect relationship that abusive supervision affects team creativity through TLMX was significant. The indirect effect was −0.276. In addition, the 95% confidence interval of the indirect relationship was [-0.606, −0.064], not containing zero. Thus, Hypothesis 1a was supported.

**TABLE 3:** Regression results for team-level analysis.

Level and variables	TLMX			Team creativity					
	Model 1	Model 2	Model 3	Model 4	Model 5	Model 6	Model 7	Model 8	Model 9
**Level 2**									
Team size	0.038	0.000	0.090	0.165	0.146	0.136	0.119	0.165	0.144
Team tenure	0.017	0.009	–0.008	–0.084	–0.088	–0.090	–0.143	–0.081	–0.091
AS	−0.476***	−0.402***	−0.588***					−0.158*	0.107
TLMX					0.506***	0.477***	0.382**		0.557***
DLMX		−0.262*	−0.281**			–0.080	–0.143		
**Interaction item**									
AS*DLMX			0.365**						
TLMX*DLMX							−0.322**		
*R*^2^	0.228	0.289	0.377	0.034	0.290	0.295	0.387	0.059	0.299
Δ*R*^2^	0.228	0.062	0.087	0.034	0.256	0.005	0.092	0.025	0.240
*F*	6.584**	6.715***	7.858***	1.185	9.113***	6.910***	8.217***	2.228*	7.026***
Δ*F*	6.584**	5.719*	9.122**	1.185	24.162***	0.503	9.771**	1.779*	22.584***

*N (leader/team) = 71. AS, abusive supervision; TLMX, team-level leader-member exchange; DLMX, LMX differentiation.* *p < 0.05; **p < 0.01; ***p < 0.001.

Hypothesis 1b proposed that abusive supervision has an indirect negative relationship with individual creativity through TLMX. As shown in Table 3, abusive supervision is negatively related to TLMX. Further, TLMX was significantly related to individual creativity (β = 0.732, *p* < 0.001; see Model 2 in Table 4). The bootstrapping test based on MacKinnon et al. (2004) procedure confirmed that the indirect relationship that abusive supervision affects individual creativity through TLMX was significant. For individual creativity, the 95% confidence interval of the indirect relationship was not containing zero. Therefore, Hypothesis 1b was supported.

**TABLE 4:** HLM results for cross-level analysis.

Level and variables	Individual creativity (DV)			
	Model 1	Model 2	Model 3	Model 4
**Level 1**				
Gender^a^	0.178*	0.169*	0.169*	0.191*
Age	0.011	0.014	0.013	0.015
Education level	0.056	0.049	0.051	0.039
LMX	0.236**	0.235**	0.238**	0.235**
**Level 2**				
Team size^b^	0.092*	0.079**	0.079**	0.078*
Team tenure	0.003	–0.005	–0.004	–0.011
Abusive supervision	−0.239*	0.063		
TLMX		0.731***	0.697***	0.671***
DLMX				–0.005
**Interaction item**				
TLMX*DLMX				–0.031

^*a*^N (subordinate) = 319. ^*b*^N (leader/team) = 71. *p < 0.05; **p < 0.01; ***p < 0.001. All coefficients in this table are unstandardized regression coefficients.

**FIGURE 3:**
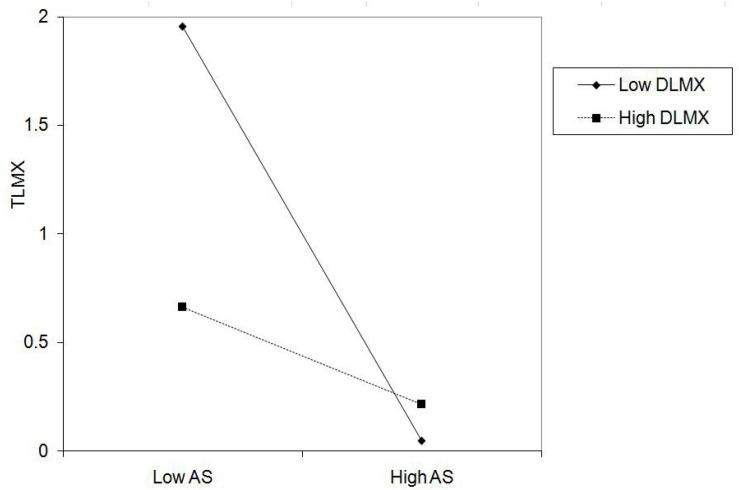
Interaction of abusive supervision and DLMX on TLMX.

Hypothesis 2 proposed that DLMX moderates the relationship between abusive supervision and TLMX such that the negative relationship is lessened when DLMX is higher. Table 3 showed that the interaction between abusive supervision and DLMX significantly affected TLMX (β = 0.365, *p* < 0.01, see Model 3 in Table 3). We further plotted the simple slopes for the relationship between abusive supervision and TLMX under high (+ 1 s.d.) and low (−1 s.d.) levels of DLMX (Aiken et al., 1991). DLMX constrained the negative relationship between abusive supervision and TLMX (see Figure 3). The results of simple slope analysis (Aiken et al., 1991) showed that the relationship between abusive supervision and TLMX was significantly negative (simple slope = −0.372, *p* < 0.01) under high DLMX, and became stronger (simple slope = −0.763, *p* < 0.001) under low DLMX. Hence, Hypothesis 2 was supported.

**FIGURE 4:**
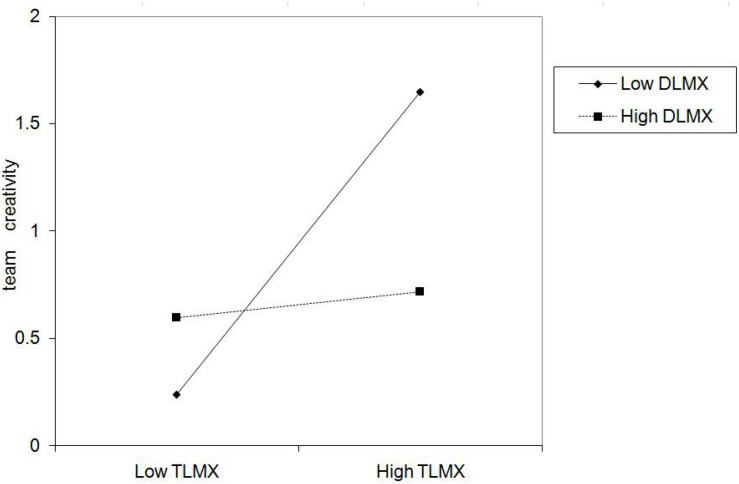
Interaction of TLMX and DLMX on team creativity.

Hypothesis 3a suggested that DLMX plays a moderating role on the relationship between TLMX and team creativity such that the positive relationship is lessened when DLMX is higher. We found that the interaction between TLMX and DLMX was negative and significant (β = −0.322, *p* < 0.01, see Model 7 of Table 3). The results of simple slope analysis (Aiken et al., 1991) revealed that the relationship between TLMX and team creativity was not significant (simple slope = 0.127, n.s) under high DLMX, and was positively significant (simple slope = 0.638, *p* < 0.001) under low DLMX (see Figure 4). Hence, Hypothesis 3a was supported.

**FIGURE 5:**
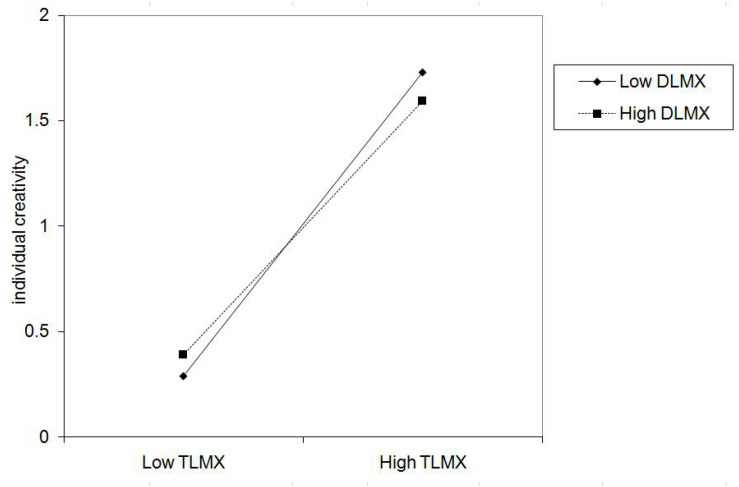
Interaction of TLMX and DLMX on individual creativity.

Hypothesis 3b suggested that DLMX moderates the positive relationship between TLMX and individual creativity. To test the cross-level hypothesis, we used HLM. In this approach, we entered DLMX as a predictor of the intercept and TLMX-individual creativity slop, while controlled for team size and team tenure. As shown in Model 4 of Table 4, there was a non-significant interaction between TLMX and DLMX (β = −0.031, n.s). Further, DLMX did not moderate the positive relationship between TLMX and individual creativity in Figure 5. Thus, Hypothesis 3b was not supported.

## Discussion and Conclusion

The purpose of this study was to delineate the relationship between abusive supervision and creativity at both team and individual levels. Based on empirical data, we found that abusive supervision negatively affects TLMX, which in turn is positively related to team creativity and individual creativity. Furthermore, DLMX played a moderating role. When DLMX is higher, the negative relationship between abusive supervision and TLMX is lessened. In addition, DLMX exhibits a moderating role on the relationship between TLMX and team creativity, such that the positive relationship is lessened when DLMX is higher.

### Theoretical Implications

This study extended previous scholarly findings on employee creativity at a single level. We examined both team creativity and individual creativity and described the mechanism through which abusive supervision affects creativity at these two levels. This study demonstrated that abusive supervision is related to both team creativity and individual creativity, and the relationships are both direct and indirect. Overall, this study offers new insight into LMX theory, multilevel theory of creativity, and abusive supervision.

It is worthwhile to highlight that the cross-level and team-level findings enrich the understanding about the consequences of abusive supervision. Different from Lee et al. (2013) who found an inverted U-shaped relationship between abusive supervision and employee creativity, this study showed that abusive supervision has negative linear relationships with both team creativity and individual creativity. Accentuating the team-level attributes of abusive supervision, our findings suggest that abusive supervision be better examined at multiple levels.

By integrating abusive supervision with LMX theory, this study regarded TLMX as a social exchange process linking behavioral antecedents and performance consequences. Given the novelty of the LMX approach to explaining creativity, little research has been done to empirically examine the underlying mechanisms. Based on well-rounded theory, we described the mechanism of how abusive supervision negatively and indirectly affects creativity through TLMX. Our empirical findings clearly answered the research call that “it would be worthwhile to test the mechanisms explaining how abusive supervision influences creative employee behaviors” (Lee et al., 2013, p. 730). As a contribution, this study offered a holistic view of the mediating role LMX takes.

Another major contribution is that we revealed the moderating role of DLMX. Our findings indicated that DLMX constrains the negative relationship between abusive supervision and TLMX (see Figures 2, 3). This finding echoed earlier studies (e.g., Li and Liao, 2014; Carnevale et al., 2019; Matta and Van Dyne, 2020). Our results suggest that when DLMX increases, the negative effect of abusive supervision will be lessened.

Another noteworthy finding is that TLMX has a weaker positive influence on team creativity under higher DLMX. Even though DLMX prompts employees to make social comparative evaluation, the social comparison process will harm team exchange process, such as information sharing (Hu et al., 2018). By examining the association between TLMX and DLMX, we were able to tease out which combinations are better suited for the promotion of creativity. Our findings indicate that the combination of a high level of TLMX and a low level of DLMX is the best circumstance for fostering team creativity. This finding provides rich insight to the research call that “further research is needed to identify contextual factors that moderate the relationship between abusive supervision and employee creativity” (Lee et al., 2013, p. 730). Moreover, our results showed that the moderating role of DLMX in TLMX-individual creativity relationship is not significant. As Matta and Van Dyne (2020) stated, “nearly all of the cross-level consequences of LMX differentiation are conditional” (p. 155).

### Practical Implications

From a managerial perspective, the findings of this study point out the negative consequences of abusive supervision in the fields of new product development and innovation management. Our findings confirmed that abusive supervision is detrimental to both team creativity and individual creativity. Thus, the findings remind organizations that abusive supervision should be avoided wherever team leaders are given chances to do so. Concerned organizations should implement training programs to educate both team leaders and team members how to prevent the occurrence of abusive supervision.

Second, teams may employ TLMX to achieve better organizational outcomes within and beyond the context of creativity. TLMX can be utilized as a functional social exchange process between the team leader and the team members. Managers should pay attention to building high-quality relationships with their subordinates before any innovative task is initiated, especially in a *guanxi* society like China.

Finally, managers should not ignore DLMX as an important contextual factor in facilitating creativity. Under lower DLMX, the positive relationship between TLMX and creativity can be strengthened. Therefore, a culture of fairness, combined with leaders’ enhanced communication skills, can help promote creativity for teams.

### Limitations and Future Research

This study is not without limitations. First, the cross-sectional research design limited the extent to which cause-effect relationships can be inferred from our findings. It is possible that abusive supervision at time 1 influences TLMX at time 2, which further enhances creativity at time 3. Therefore, a longitudinal study should be conducted to better analyze the chronological and sequential effects of abusive supervision on creativity.

Second, our study only focused on examining TLMX as a social exchange process linking abusive supervision and team creativity. Other potential team-based work processes, such as team learning and knowledge sharing, may have taken effects as well. Hence, future research should examine what roles other team processes play at the same time.

Third, we regarded abusive supervision as a team-level attribute by aggregating individual perception on abusive supervision. We did not test if the team leader’s self-rating of abusive supervision is consistent with the team members’ ratings. Therefore, for the purpose of verification and controlling social desirability, future research should consider the agreement or disagreement of abusive supervision between the team leader and the team members.

Lastly, this study was conducted in China, a country where individuals have relatively high power distance and high collectivism. This may limit our findings’ generalizability to teams in Western countries. Thus, future research may conduct cross-cultural comparisons to see if culture influences the relationships and parameters identified in this study.

## Data Availability Statement

The original contributions presented in the study are included in the article/supplementary material, further inquiries can be directed to the corresponding author/s.

## Author Contributions

CH, LZ, and VW conceptualized the research idea. RT and JY collected and analyzed the data. CH wrote the initial draft of the manuscript. LZ reviewed and confirmed the final manuscript. All authors read and agreed to the published version of the manuscript.

## Conflict of Interest

The authors declare that the research was conducted in the absence of any commercial or financial relationships that could be construed as a potential conflict of interest.
